# Supplementary material for: Content-rich biological network constructed by mining PubMed abstracts
Source: BMC Bioinformatics. 2004 Oct 8;5:147. doi: 10.1186/1471-2105-5-147 (PMC528731; doi:10.1186/1471-2105-5-147)
Supplement: Additional File 2 — The original results of the above study (non-essential files are deleted to keep the file size under the limit set by BMC bioinformatics). [file 1471-2105-5-147-S2.bz2 › chilibotAdditionalFile2/dip10/639ID10966114E11026/html/CBL_UBE2L3.html]

 


 **CBL** and **UBE2L3** 
  
Found 14 abstracts in PubMed, retrieved 10.  
 

 What does Google say? 
 PDF only 
| .edu only 

---

**Interactive relationship** (e.g. stimulation, inhibition, etc)

**Neutral relationship**- We have also established that the RING1 finger of HHARI cannot be substituted for by the highly homologous RING finger domains of either of the ubiquitin protein ligase  [ **UBE2L3** ]  components c  **CBL**  or Parkin, despite their similarity in structure and their independent capabilities to bind UbcH7  [ **UBE2L3** ] .  Ref: 11278816 J Biol Chem, 2001
- hSpry2 interacts specifically with the c  **Cbl**  RING finger domain and displaces UbcH7  [ **UBE2L3** ]  from its binding site on the E3 ligase.  Ref: 12234920 EMBO J, 2002
- Our results suggest that the ubiquitin protein ligase  [ **UBE2L3** ]  function of  **Cbl**  b is regulated by coordinated degradation of the  **Cbl**  b protein along with its substrate.  Ref: 11375397 J Biol Chem, 2001
- In vitro, c  **Cbl**  forms a stable complex with the ubiquitin conjugating enzyme UbcH7  [ **UBE2L3** ] , but active Src destabilizes this interaction.  Ref: 11448952 J Biol Chem, 2001
- The RING finger domain of c  **Cbl**  has been shown recently to enhance ubiquitination of active RTKs by acting as an E3 ubiquitin protein ligase  [ **UBE2L3** ] .  Ref: 11071924 Mol Biol Cell, 2000

**Non-interactive relationship** (e.g. studied together, co-existance, homology, etc.)

- Here we report biochemical and genetic characterization of the Drosophila homologue of c  **Cbl** , a negative regulator of signal transduction with ubiquitin protein ligase  [ **UBE2L3** ]  activity.  Ref: 10918586 Oncogene, 2000
- Here we report that  **Cbl** , a RING type E3 ubiquitin protein ligase  [ **UBE2L3** ] , promotes ubiquitination of TCR zeta chain, which requires its functional variant Src homology 2 domain and an intact RING finger.  Ref: 11353765 J Biol Chem, 2001
- Structure of a c  **Cbl**  UbcH7  [ **UBE2L3** ]  complex RING domain function in ubiquitin protein ligases.  Ref: 10966114 Cell, 2000
- **Cbl**  b, a ring type E3 ubiquitin protein ligase  [ **UBE2L3** ] , is implicated in setting the threshold of T lymphocyte activation.  Ref: 11526404 Nat Immunol, 2001
- Recently, evidence has started to emerge that the ubiquitin protein ligase  [ **UBE2L3** ]  c  **Cbl**  is involved in CSF 1 receptor degradation.  Ref: 11850825 Oncogene, 2002
